# Editorial: Nutritional status in disease: development, evaluation, prognosis and individualized treatment in clinical practice

**DOI:** 10.3389/fnut.2023.1292777

**Published:** 2023-10-09

**Authors:** Wei Sang

**Affiliations:** Department of Hematology, Affiliated Hospital of Xuzhou Medical University, Xuzhou, Jiangsu, China

**Keywords:** individualized treatment, nutritional status, prognosis, development, clinical practice

This Research Topic focuses on the association between nutritional status and clinical outcomes of diseases, including disease occurrence, prognosis, and personalized treatment. In this thematic organization, four clinical experts from different medical fields were invited to collaborate on this task and put in diligent work. After careful screening and selection, 22 excellent clinical research results on this specific topic were finally presented. The Research Topic emphasizes nutrition and incorporates various nutritional assessment systems, metabolic parameters related to nutrition, and other valuable nutritional evaluation indicators. The clinical studies cover a wide range of clinical disease states, including tumors, metabolic and rheumatic diseases, surgical diseases, infections, and provide comprehensive and insightful descriptions of nutritional indicators.

The nutritional status has an impact on the prognosis and clinical outcomes of solid tumors, as well as on patient treatment adherence. It is also an important consideration factor for guiding personalized treatment for cancer patients. Ge et al. explored the prognostic value of the Prognostic Nutritional Index (PNI) in follicular lymphoma (FL) and confirmed the correlation between PNI and survival in FL. PNI was identified as the sole predictive factor in the multivariate analysis for overall survival (OS). Moreover, there is potential for integrating PNI into the FLIPI prognostic system to achieve more accurate prognostic stratification for FL. Shen et al. used visceral fat area (VFA) as an entry point to evaluate nutritional status in Diffuse large B-cell lymphoma (DLBCL). They included fat content at the third lumbar vertebra and albumin level as research variables. Through X-Tile analysis, they identified a VFA cutoff value for prognostic differentiation. The study confirmed that VFA can stratify the prognosis of DLBCL, and a model (model2) based on VFA, along with other variables such as albumin, ki67, and β2-MG, also demonstrated prognostic stratification value for specific DLBCL subtypes. Immunodeficiency Virus (HIV)-related lymphomas have specific clinical and pathological characteristics, but the impact of nutritional status on the prognosis of HIV-related lymphomas remains unknown. Liu et al. incorporated the CONUT nutritional assessment system and analyzed the prognosis of 149 patients with HIV-related lymphoma. The results of the multivariate analysis confirmed that CONUT, along with other relevant factors, is an independent risk factor affecting patient prognosis. This suggests that strengthening nutritional support is of significant importance in the treatment of HIV-related lymphomas. Amino acids are important components involved in the three major metabolism pathways, influencing energy supply, redox balance, and other regulatory mechanisms such as epigenetics and tumor microenvironment. Wang et al. discussed the impact of different amino acid metabolism on tumor cells and proposed that combining amino acid starvation therapy with conventional chemotherapy could potentially overcome chemotherapy resistance and achieve higher response rates. Zhou et al. screened 472 amino acid metabolism-related genes and identified six candidate genes to develop a risk prediction model. They confirmed that this model is associated with the tumor immune microenvironment (TME) of acute myeloid leukemia (AML), aiding in predicting AML prognosis and the efficacy of immunotherapy. Li et al. demonstrated that in hospitalized esophageal cancer patients receiving chemotherapy, oral nutritional supplements, although increasing hospital costs, are beneficial in reducing the incidence of bone marrow suppression, particularly in patients with a BMI ≤ 18.5 kg/m^2^.

Immunological and metabolic diseases often lead to chronic clinical processes that require long-term specialized and supportive treatment. This Research Topic includes several clinical studies exploring the relationship between nutrition and these two types of chronic diseases. Zhang et al. investigated the impact of the advanced lung cancer inflammation index (ALI) on the prognosis of hypertension. They used ALI for prognostic grouping and found that higher ALI scores were associated with increased long-term all-cause mortality in hypertensive patients. Tian et al. collected data from 1,967 rheumatoid arthritis (RA) patients of the National Health and Nutrition Examination Survey (NHANES) to evaluate the relationship between nutritional indicators (CONUT/NRI) and prognosis. The results confirmed that a certain proportion of RA patients had malnutrition, which increased the risk of death. This study emphasized the impact of nutritional status on the prognosis of RA patients. In addition, a certain proportion of RA patients experience muscle atrophy. Pan et al. explored the prevalence of early RA combined with muscle atrophy and its impact on survival. The study showed that early RA patients had a higher prevalence of sarcopenia, as well as higher disease activity scores and rates of physical functional impairment compared to healthy individuals. This highlights the importance of early disease control in preventing early muscle atrophy and provides new insights for the clinical nutritional management of RA. Type 2 diabetes mellitus (T2DM) is a chronic metabolic disease, and simultaneous control of blood glucose and prevention of complications are the main treatment strategies. Song et al. included the A2DS2 nutritional assessment system and other relevant variables to develop a predictive model for Stroke-associated pneumonia (SAP) in T2DM patients. They proposed that the A2DS2 model could be a reliable predictive model for SAP in T2DM patients. Barisic et al. shared valuable clinical treatment experience in a case report of gastrointestinal myopathy. Considering the presence of intestinal motility disorders and dysbiosis, the study emphasized the importance of nutritional support in clinical practice, which led to better treatment outcomes for the patient. Zhu et al. explored the prognostic correlation between blood lipids and benign prostatic hyperplasia (BPH). By analyzing the correlation and prognosis of healthy males and BPH patients, the study suggested that blood lipids are an independent risk factor for BPH and should be controlled. Skeletal muscle mass index (SMI) is a quantitative indicator reflecting muscle nutritional status. Bai et al. studied the predictive value of SMI for acute-on-chronic liver failure. The results indicated that higher SMI is an independent protective factor for acute-on-chronic liver failure. Long-term treatment outcomes of ischemic stroke are influenced by multiple factors, and dietary intervention is an important component of supportive therapy. Besseau et al. demonstrated that dietary interventions during hospitalization are beneficial for improving the long-term dietary habits of ischemic stroke patients. Nutritional support for chronic diseases affects the occurrence of patient complications. Bong et al. studied the occurrence of pressure ulcers and changes in albumin levels in patients referred to a nutrition support team (NST). The results confirmed that NST nutritional therapy plays a significant role in reducing pressure ulcers, particularly in elderly patients. Dong et al. demonstrated that combined folic acid and enalapril can better reduce homocysteine levels compared to monotherapy with enalapril alone. This subsequently reduces the occurrence of atrial fibrillation after radiofrequency ablation in patients with hypertensive atrial fibrillation.

Clinical surgical problems and the treatment of infectious diseases often rely on nutritional support. Ma et al. conducted a nutritional assessment of critically ill burn patients, incorporating the Modified Nutrition Risk in Critically ill (mNUTRIC) and Nutrition Risk Screening 2002 (NRS2002) tools. They analyzed the predictive ability of these two indicators for patient mortality and the factors contributing to 28-day mortality in severe burn patients. The study demonstrated the effectiveness of mNUTRIC as a nutritional risk screening tool for severe burn patients.

Timely postoperative recovery of food intake and gastrointestinal nutritional support are crucial for the rehabilitation of patients after abdominal surgery. Nguyen et al. conducted a dynamic analysis of postoperative anorexia in abdominal surgery patients and assessed it using the Council on Nutrition Appetite Questionnaire (CNAQ). The study found that changes in appetite within 7 days after surgery were significant. Interventions to address postoperative appetite decline should be strengthened in patients undergoing abdominal surgery under general anesthesia and laparotomy. Cai et al. evaluated the predictive value of the CONUT nutritional assessment system for postoperative complications in non-expanded lung resections in patients with bronchiectasis. The study demonstrated that CONUT can predict postoperative complications in patients with bronchiectasis after lung resection. Gong et al. included four nutritional screening tools to assess their impact on the short-term prognosis of orthopedic and neurosurgical patients. The results showed that the Geriatric Nutritional Risk Index (GNRI) had better predictive value for the short-term prognosis of elderly orthopedic and neurosurgical patients in the perioperative period. COVID-19 infection can lead to severe complications, and prognostic assessment of critically ill patients with COVID-19 helps guide individualized treatment. Zhou et al. retrospectively analyzed the correlation between nutrition and prognosis in critically ill patients with COVID-19 using the NRS2002 scoring system. The results demonstrated that early nutritional intervention can help reduce the occurrence of critical illness and improve prognosis. Yu et al. analyzed a large population and demonstrated the clinical value of serum 25-hydroxyvitamin D in the treatment of osteoarthritis.

In conclusion, the aforementioned studies have conducted research on nutritional-related prognostic risks and correlations in various clinical issues, including surgery, infection, cancer, and metabolic diseases. They emphasize the value of nutrition in the precise assessment of diseases, supportive treatment, prognostic judgment, and individualized therapy.

## Author contributions

WS: Writing—original draft, Writing—review and editing.

